# Encoding metabolic stress into tumor evolution through ECM–integrin-dependent transcriptional plasticity

**DOI:** 10.1016/j.redox.2026.104467

**Published:** 2026-11-02

**Authors:** Zixing Meng, Xin He, Mengyang Li, Chengsheng Wu

**Affiliations:** a College of Life Sciences, University of Chinese Academy of Sciences, Beijing, 101408, China; b Faculty of Hepatopancreatobiliary Surgery, The First Medical Center, Chinese People's, Liberation Army General Hospital, Beijing, 100039, China

**Keywords:** Metabolic stress, Extracellular matrix, Integrin, Transcriptional plasticity, Tumor evolution

## Abstract

Tumor evolution is driven not only by genetic alterations but also by the capacity of cancer cells to adapt to dynamic microenvironmental stresses. Emerging evidence indicates that metabolic constraints can remodel the extracellular matrix (ECM), extending stress adaptation beyond cell-intrinsic programs. Importantly, stress-induced changes in ECM composition, organization and mechanics may persist beyond the initiating stimulus and influence subsequent cell–matrix signaling. We refer to this proposed persistence of stress-associated extracellular changes as“ECM encoding”, a form of physicochemical information storage that is mechanistically distinct from genetic memory or cell-intrinsic epigenetic stabilization. Here, we critically review the ECM–integrin axis as a signaling interface linking metabolic stress, redox adaptation and transcriptional plasticity. Metabolic pressures, including hypoxia, nutrient limitation and isolation stress, reshape ECM composition, mechanics and ligand availability, thereby reprogramming integrin signaling. Through focal adhesions, cytoskeletal tension and nuclear mechanotransduction, integrin-dependent signals can influence chromatin accessibility and converge with stress-responsive networks involving YAP/TAZ, ATF4, HIF, NRF2, STAT3 and NF-κB. In parallel, matrix-dependent regulation of mitochondrial reactive oxygen species production and NRF2-mediated antioxidant responses may provide mechanisms linking extracellular cues to redox adaptation. These interconnected processes can promote phenotypic plasticity associated with stemness, dormancy, metastasis, immune evasion and therapeutic resistance, although their relative contributions vary across tumor types and experimental contexts. Finally, we evaluate the therapeutic potential of targeting the ECM–integrin–redox network while considering signaling redundancy, previous clinical setbacks, and the need for biomarker-guided patient selection.

## Highlights

• Metabolic stress reshapes ECM composition, mechanics, and integrin signaling.
• ECM–integrin signaling links redox adaptation to transcriptional plasticity.
• Stress-adapted stromal and immune cells reshape tumor microenvironments.
• Targeting ECM–integrin dependencies may help overcome therapeutic resistance.

## 1 Introduction

Cancer progression is shaped by both malignant cells and their surrounding ecosystem, which includes fibroblasts, immune cells, endothelial cells, and a dynamically remodeled extracellular matrix (ECM) [1]. Aberrant vascular perfusion and imbalanced nutrient consumption generate metabolically stressed niches, further compounded by acidosis, oxidative stress, lipid imbalance, and therapy-induced damage [2]. These conditions select for plastic, stress-tolerant, and therapy-adapted tumor cell states [3].

While prior studies have emphasized intracellular stress-sensing pathways, such as the integrated stress response, NRF2-mediated antioxidant defense, and hypoxia-inducible factor (HIF) signaling, emerging evidence indicates that metabolic stress also modifies the extracellular landscape [4], [5]. The tumor-associated ECM undergoes structural and mechanical alterations, including collagen crosslinking, fibronectin accumulation, and increased stiffness, thereby reshaping how cells interpret their microenvironment [6].

Integrins, as key ECM receptors, transduce these external cues into intracellular signals through FAK, SRC, PI3K–AKT, and RhoA–ROCK pathways [7]. Beyond adhesion, integrin-mediated mechanotransduction regulates nuclear architecture and chromatin accessibility, linking physical stress to transcriptional plasticity [8]. Recent findings suggest that integrins are not merely passive responders; their expression may also be induced under metabolic stress, or integrin-expressing cells may be selectively enriched, enabling integrin-dependent regulation of nutrient acquisition, survival signaling, and immune modulation [9], [10], [11], [12].

Here, we use the term “ECM encoding” to describe stress-induced changes in ECM composition, architecture, mechanics and ligand accessibility that may persist beyond the initiating stress and thereby alter subsequent integrin-mediated signaling. This concept does not imply genetic memory or autonomous epigenetic inheritance, and the persistence and reversibility of such ECM states are expected to vary across tumor types and microenvironmental contexts. In this Review, we examine a conceptual framework in which the ECM–integrin axis links metabolic stress with transcriptional plasticity and may contribute to tumor adaptation and therapeutic response. We summarize how stress reshapes ECM composition, how integrins sense these changes, and how resulting adhesion signals reprogram transcription to support tumor adaptation and therapeutic response. This perspective highlights integrins as central stress-responsive regulators and potential therapeutic targets, offering opportunities to disrupt tumor resilience beyond targeting metabolic enzymes or isolated transcription factors.

## 2 Metabolic stress remodels ECM–integrin niches

Metabolic stress is not restricted to intracellular nutrient sensing or energy homeostasis [4]. In solid tumors, heterogeneous oxygen availability, nutrient limitation, acidosis, redox imbalance and isolation stress emerge from abnormal perfusion, high metabolic demand and dynamic stromal remodeling [2], [5]. These pressures do not simply challenge tumor cell survival; they reshape the ECM landscape, altering matrix composition, mechanical properties and ligand availability [13]. These ECM changes can modify integrin engagement and reinforce adaptive signaling initiated by metabolic stress [7](Fig. 1).

**Fig. 1:**
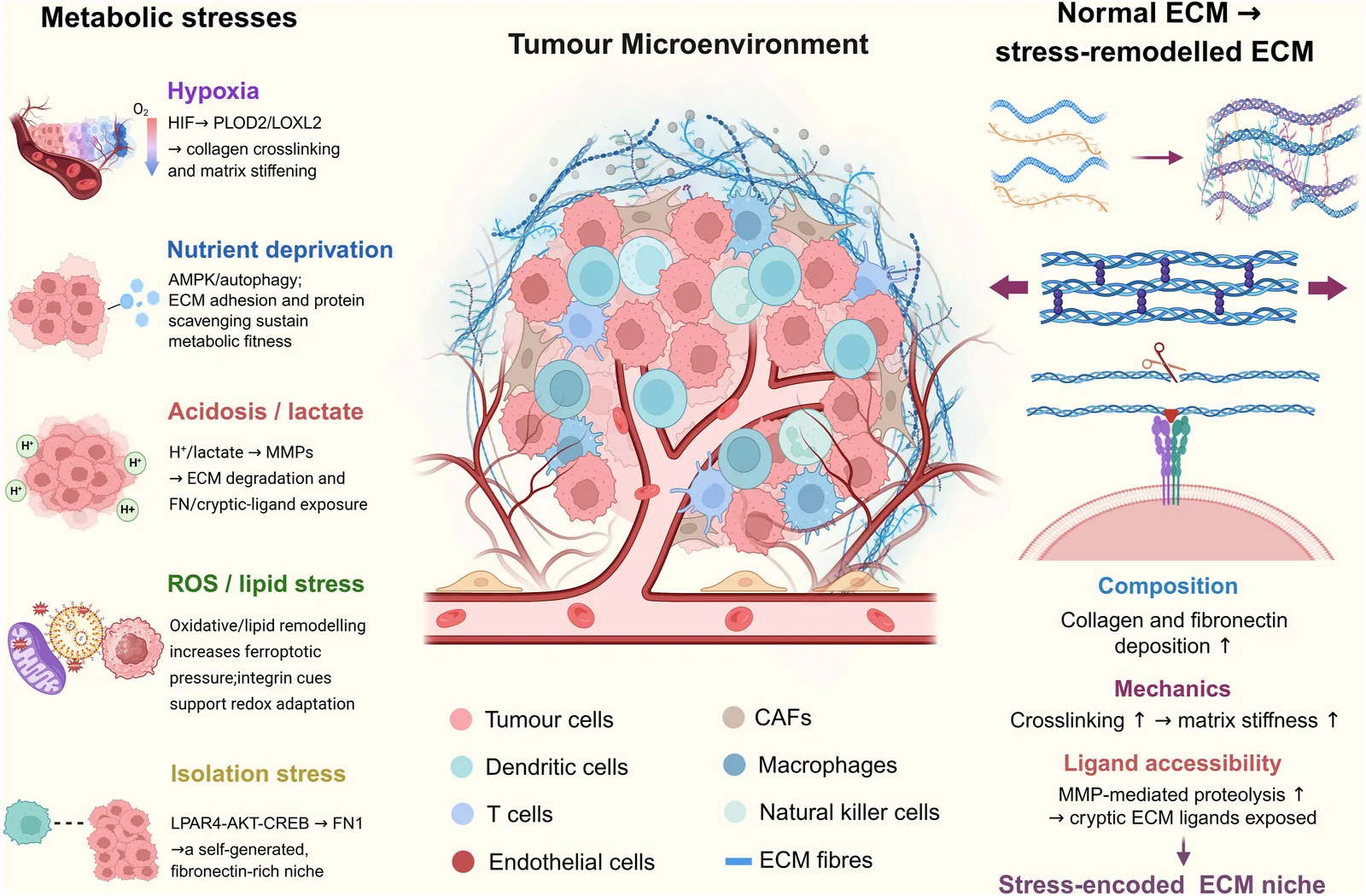
**Metabolic stress-associated ECM remodeling shapes context-dependent integrin signaling in tumor niches.** Solid tumors experience spatially heterogeneous metabolic stresses, including hypoxia, nutrient deprivation, acidosis and lactate accumulation, reactive oxygen species (ROS) and lipid stress, and isolation stress. These pressures can influence tumor-cell adaptation and ECM remodeling through distinct, potentially interconnected mechanisms. Representative processes include HIF-associated regulation of PLOD2 and LOXL2, AMPK–autophagy signaling, MMP-mediated ECM proteolysis and integrin-associated redox adaptation. LPAR4-associated formation of fibronectin-rich niches illustrates an additional, context-specific mechanism of ECM remodeling. Depending on the tumor and microenvironmental context, these processes may contribute to changes in ECM composition, mechanical properties and ligand accessibility, including collagen and fibronectin deposition, collagen crosslinking, matrix stiffening, proteolysis and exposure of cryptic ECM ligands. The resulting matrix alterations can provide biochemical and mechanical cues that influence integrin-associated signaling and tumor-cell adaptation. These processes represent parallel and potentially interconnected mechanisms rather than a single, universally established signaling sequence.

### 2.1 Hypoxia-driven ECM remodeling and integrin reprogramming

Hypoxia is a dominant feature of solid tumors and is traditionally viewed as a trigger of HIF-dependent metabolic adaptation [5]. However, hypoxia also acts as an ECM-organizing stress by altering collagen architecture, fibronectin deposition and integrin expression [14], [15]. HIF-regulated collagen-modifying enzymes, including PLOD2 and LOXL2, influence collagen maturation, crosslinking and tissue mechanics, with evidence derived from both tumor and fibrosis models [16], [17]. In a sarcoma model, hypoxia-induced PLOD2 modifies collagen VI and weakens the lung endothelial barrier; in uveal melanoma models, HIF-regulated collagen hydroxylation supports collagen deposition and tumor growth [14], [17].

Hypoxia also reshapes the ECM receptor landscape of tumor cells. In a breast cancer model, HIF-dependent induction of integrins, including α5β1 and α6-containing integrins, enhances tumor cell interactions with remodeled ECM and promotes migration, invasion, and tumor initiation [15], [18]. In pancreatic ductal adenocarcinoma (PDAC), hypoxia additionally increases αvβ3 integrin expression through STAT3-dependent regulation, further reinforcing adhesion-dependent survival [19]. Thus, hypoxia modifies both sides of the ECM–integrin interface by restructuring matrix ligands and favoring integrin expression programs that support stress adaptation.

### 2.2 Nutrient stress and ECM-dependent metabolic adaptation

Nutrient deprivation arises from abnormal vascular supply and competition among tumor, stromal and immune cells [2]. Although nutrient stress activates intracellular pathways such as AMPK, mTOR and the integrated stress response, ECM engagement provides an additional survival input by maintaining metabolic fitness [4], [9]. Loss of matrix attachment reduces glucose uptake, ATP production and redox buffering, increasing vulnerability to anoikis [20].

Under nutrient limitation, integrin signaling can actively promote metabolic flexibility. For example, stress-induced αvβ3 expression sustains SRC–AMPK–PGC-1α signaling, supporting mitochondrial adaptation and glutamine-dependent metabolism in lung cancer cells even under anchorage-independent conditions [9]. Moreover, the ECM itself can serve as a metabolic reservoir. Invasive cancer cells can internalize and degrade extracellular proteins through mechanisms such as macropinocytosis and subsequently degrade them through lysosomal pathways to obtain alternative nutrients [21], [22]. Together, integrin signaling and ECM-derived nutrients can support metabolic adaptation through complementary mechanisms.

### 2.3 Acidosis and redox stress reshape adhesion landscapes

Acidosis represents another metabolic pressure that directly modifies ECM–integrin interactions [23], [24]. Elevated glycolysis, lactate accumulation and impaired proton clearance generate acidic niches that promote ECM degradation, protease activation and exposure of cryptic matrix ligands [25], [26]. These changes alter integrin engagement and facilitate invasive behavior. Acidic extracellular conditions can also enhance integrin activation, linking pH adaptation with focal adhesion signaling and migration.

Redox and lipid stress further illustrate the reciprocal relationship between matrix cues and tumor metabolism. Experimental studies indicate that ECM attachment and integrin signaling regulate mitochondrial function, ROS buffering, and ferroptotic susceptibility [27]. In cultured breast cancer cells, α6β4–SRC–STAT3 signaling suppresses ACSL4 expression, limiting lipid peroxidation and ferroptosis. In lung adenocarcinoma, cell culture and mouse studies link increased matrix stiffness to ferroptosis resistance through integrin–YAP-associated signaling, whereas human evidence remains correlative [28], [29]. Soft matrices can promote mitochondrial fission and mitochondrial ROS–NRF2 signaling, supporting antioxidant adaptation in cultured breast cancer cells [30]. Conversely, in cultured triple-negative breast cancer cells, ACSL4-dependent membrane phospholipid remodeling promotes integrin β1 activation, providing cellular evidence for reciprocal regulation between lipid metabolism and adhesion [31].

Together, these findings suggest that redox regulation at the ECM–integrin interface is bidirectional. ECM mechanics and integrin engagement can influence mitochondrial ROS production and antioxidant responses, whereas redox and lipid remodeling can in turn modulate integrin activation and adhesion signaling. In parallel, oxidative stress may affect ECM remodeling by altering matrix deposition, modification and proteolytic turnover, thereby changing the extracellular cues subsequently sensed by tumor cells. The relative importance of these reciprocal mechanisms is likely to depend on tumor type, metabolic state and microenvironmental context.

### 2.4 Isolation stress and a cell-autonomous fibronectin-rich niche

Isolation stress provides a distinct example of how tumor cells actively construct ECM-dependent survival niches. During dissemination or early tumor initiation, isolated tumor cells experience loss of cell–cell contacts, reduced stromal support, and increased susceptibility to anoikis [32]. In PDAC, isolation stress induces LPAR4–AKT–CREB signaling, leading to a tumor-cell-generated fibronectin-rich niche. Fibronectin engagement through integrins α5β1 and αvβ3 subsequently reinforces survival signaling and enables neighboring tumor cells to acquire stemness and stress tolerance [10].

Additional studies identified the NF-κB family member c-Rel as another regulator of fibronectin–integrin adaptation in PDAC, where c-Rel-dependent induction of fibronectin and integrin β3 promotes anchorage-independent growth and metastatic colonization [11]. Together, these findings show how tumor-cell-derived ECM can reinforce adaptation to isolation stress in PDAC. Whether this niche-dependent support persists after stress withdrawal or whether it is accompanied by cell-intrinsic epigenetic stabilization, requires further investigation.

## 3 ECM–integrin signaling drives transcriptional adaptation to metabolic stress

Metabolic stress-induced ECM remodeling alters the adhesive and mechanical information transmitted through integrins, thereby influencing tumor cell transcriptional responses to stress [7], [13]. Changes in collagen architecture, fibronectin deposition, matrix stiffness and ligand availability reshape how tumor cells interact with their niches and influence the magnitude and duration of downstream survival signals [6]. Thus, ECM–integrin signaling does not replace canonical intracellular stress sensors; rather, HIF and AMPK signaling, the integrated stress response and other cell-intrinsic pathways often initiate acute responses to metabolic perturbation, whereas ECM–integrin signaling may subsequently amplify, redirect or prolong selected adaptive programs by modifying adhesive and mechanical inputs.

Integrins represent a major interface through which remodeled ECM influences intracellular adaptation [7]. Ligand engagement induces integrin clustering and focal adhesion assembly, coupling extracellular cues to FAK/SRC signaling, actomyosin contractility and nuclear mechanotransduction [7]. These processes connect ECM remodeling with transcriptional regulation through two interconnected mechanisms: biochemical signaling cascades that activate stress-responsive transcription factors, and mechanical forces that reshape nuclear architecture and chromatin accessibility [8], [33]. Through these events, integrin signaling can help tumor cells survive and maintain phenotypic plasticity and stress tolerance under metabolically adverse conditions [12](Fig. 2).

**Fig. 2:**
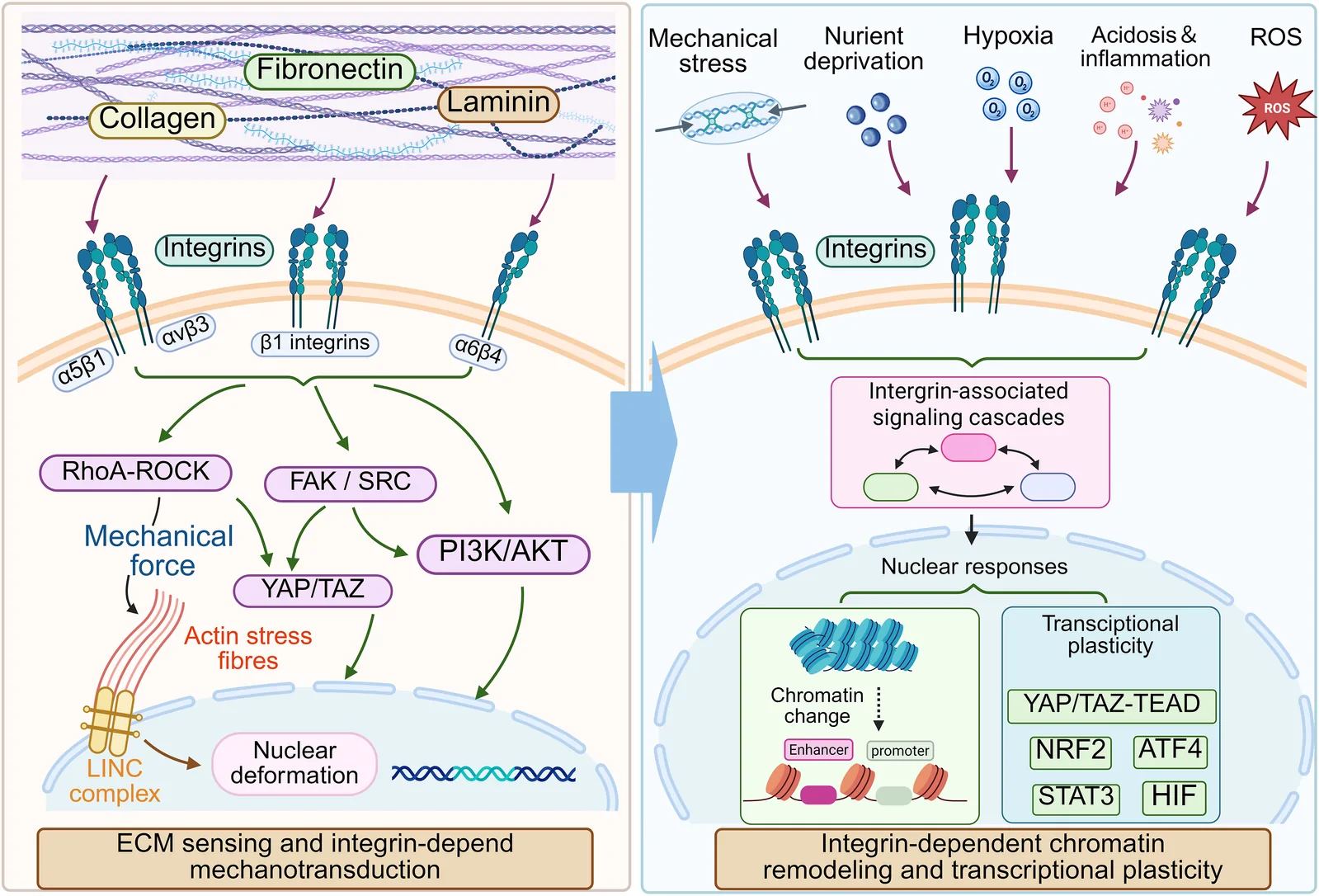
**ECM–integrin-associated signaling links extracellular stress sensing and mechanotransduction to chromatin accessibility and transcriptional plasticity.** ECM components, including collagen, fibronectin and laminin, engage integrin receptors on tumor cells and activate context-dependent signaling pathways involving RhoA–ROCK, FAK/SRC and PI3K/AKT. Integrin-mediated adhesion and cytoskeletal tension can influence nuclear mechanics through the linker of nucleoskeleton and cytoskeleton (LINC) complex, potentially affecting nuclear architecture and chromatin organization. In parallel, integrin-associated signaling can regulate mechanosensitive transcriptional regulators, including YAP/TAZ. Diverse microenvironmental stresses, including mechanical stress, nutrient deprivation, hypoxia, acidosis, inflammation and oxidative stress, may further modulate integrin-associated signaling or independently activate stress-responsive pathways involving ATF4, HIF, NRF2 and STAT3. These biochemical, mechanical and metabolic signals can influence chromatin accessibility and transcriptional programs through distinct but potentially interconnected mechanisms, with their relative contributions varying across cellular and microenvironmental contexts. The schematic illustrates these parallel and context-dependent regulatory interactions rather than a single, universally established signaling sequence.

### 3.1 ECM–integrin mechanotransduction reshapes chromatin accessibility

Metabolic stress-associated ECM changes influence transcriptional competence by regulating nuclear mechanics and chromatin organization [8]. Integrin activation promotes Rho–ROCK-dependent actomyosin tension, which is transmitted to the nucleus through the linker of nucleoskeleton and cytoskeleton (LINC) complex [34]. In parallel, FAK/SRC signaling regulates downstream transcription factors and other regulators of gene expression [7].

The mechanical properties of the ECM can directly influence chromatin states [35]. Increased matrix stiffness induces nuclear deformation, alters lamina-associated chromatin organization and increases chromatin accessibility at regulatory regions enriched for mechanosensitive transcription factor binding [8], [34]. However, these effects are highly context dependent: stiff matrices promote YAP/TAZ- and Sp1-associated programs in some epithelial models, whereas soft matrices can activate distinct lineage-specific regulatory elements in other tumor contexts [36]. These findings indicate that ECM mechanics do not impose a universal transcriptional outcome; rather, they define a regulatory landscape in which stress-responsive transcription factors act.

Emerging evidence indicates that integrin-linked pathways provide a convergence point between ECM mechanics and metabolic stress-responsive chromatin regulation. For example, hypoxia and matrix stiffness cooperatively enhance integrin β1 and integrin-linked kinase (ILK) signaling, promoting stem-like phenotypes through PI3K–AKT activation [37]. Therefore, stress-remodeled ECM may influence chromatin accessibility in ways that support subsequent transcriptional adaptation.

### 3.2 Stress-responsive transcriptional hubs linked to ECM–integrin signaling

During adaptation to microenvironmental stress, multiple transcriptional hubs integrate mechanical, metabolic and inflammatory inputs. Rather than functioning as independent pathways, YAP/TAZ, ATF4, HIF, NRF2, STAT3 and NF-κB form interconnected regulatory networks that both respond to and reinforce ECM–integrin-dependent adaptation [4], [38], [39].

➢ YAP/TAZ: mechanotranscriptional interpreters of ECM stress

YAP/TAZ are major transcriptional effectors of ECM-dependent mechanotransduction, linking integrin-associated signaling and cytoskeletal tension to TEAD-dependent gene expression [38], [40]. Metabolic stress can modify this coupling rather than uniformly activating YAP/TAZ. In YAP-dependent cancer models, biguanide treatment under glucose restriction depletes ATP, disrupts focal adhesions and suppresses FAK-associated signaling, leading to RhoA–LATS-dependent inhibition of YAP/TAZ. Restoration of ATP levels through fructose supplementation rescues FAK and YAP/TAZ activity, while constitutively active RhoA or LATS1/2 depletion counteracts YAP/TAZ phosphorylation [41]. This provides an example of energy stress altering the adhesion-associated signaling machinery upstream of YAP/TAZ, although it does not establish altered expression of a specific integrin heterodimer. Conversely, inhibition of glutamine utilization activates YAP in lung cancer models through reduced cAMP–PKA-dependent LATS phosphorylation. YAP-dependent CTGF secretion then stimulates surrounding fibroblasts to produce fibronectin and collagen [42]. Thus, metabolic conditions can reshape adhesion-associated YAP/TAZ activity, while YAP can modify ECM composition and extracellular ligand availability, thereby influencing subsequent integrin signaling.

➢ ATF4, HIF and NRF2: integrating nutrient, oxygen and redox stress

ATF4, HIF and NRF2 regulate major metabolic stress-responsive transcriptional programs that cooperate with ECM–integrin signaling [4], [5], [39].

ATF4 connects loss of matrix attachment with intracellular stress adaptation and, in stromal cells, with ECM production. In detached prostate cancer cells, ATF4 promotes CEMIP transcription, enabling PKCα-dependent BCL2 phosphorylation and the release of BECN1 to support protective autophagy and anoikis resistance [43]. This places an ATF4-dependent survival program downstream of altered cell–matrix attachment, although the study does not identify a specific integrin as the initiating receptor. In stromal cells, ATF4 supports type I collagen biosynthesis by regulating COL1A1 expression and the availability of glycine and proline. Host or fibroblast-specific Atf4 deletion impairs collagen deposition, perivascular CAF activation and tumor vascularization in melanoma and pancreatic cancer models [44]. These observations identify complementary relationships between matrix attachment, ATF4-dependent survival and stromal matrix production. ATF4 therefore occupies distinct positions within the matrix-adaptation axis, functioning downstream of attachment loss in tumor cells while promoting collagen production in stromal cells.

HIF signaling provides another connection between oxygen limitation and adhesion adaptation [5]. In addition to inducing metabolic and angiogenic programs, hypoxia can modify integrin activity and cell migratory behavior. In breast and head and neck cancer models, hypoxia/HIF signaling promotes calpain-2-dependent talin-1 cleavage, reduces integrin β1 activity and favors amoeboid migration with reduced adhesion [45]. Conversely, in hepatocellular carcinoma models, increased matrix stiffness engages an integrin β1–Piezo1 pathway that promotes Ca^2+^ influx and reduces HIF-1α ubiquitination. The resulting HIF-1α accumulation supports the expression of pro-angiogenic factors, including VEGF, CXCL16 and IGFBP2 [46]. Thus, HIF can alter the adhesive machinery, whereas integrin-associated mechanosignaling can modify HIF-1α protein stability. These mechanisms demonstrate bidirectional regulation across defined tumor contexts; they do not imply that hypoxia invariably increases integrin activity or that the HIF-1α mechanism extends to HIF-2α.

NRF2 links matrix-dependent redox adaptation to the regulation of the adhesive phenotype. In a breast cancer model, a soft ECM and reduced actomyosin tension favor DRP1-dependent mitochondrial fission, mitochondrial ROS production and NRF2-mediated antioxidant adaptation. Inhibiting DRP1 or NRF2 restores cisplatin sensitivity in disseminated cells residing in the soft lung environment [30]. This establishes a matrix-mechanics-to-NRF2 connection without assigning the response to a specific integrin heterodimer. Conversely, in radioresistant triple-negative breast cancer cells, NRF2 promotes the transcription of HNRNPL, which supports circular RNA-mediated sequestration of let-7 microRNAs and stabilization of ITGB3 transcripts, increasing integrin β3 expression and invasive behavior [47]. A two-way connection therefore emerges between matrix mechanics and NRF2-dependent adaptation, with extracellular mechanics shaping NRF2 activity and NRF2, in turn, influencing integrin expression.

➢ STAT3 and NF-κB: linking inflammation and ECM adaptation

Inflammatory transcription factors connect extracellular stress with integrin regulation. In PDAC, hypoxia and inflammatory cytokines activate a STAT3-dependent ITGB3 program that promotes stress tolerance and tumor initiation [19]. In colorectal cancer models, dysadherin activates integrin–FAK–STAT3 signaling to induce carbonic anhydrase IX, supporting pH regulation and adaptation to an acidic microenvironment [24]. Studies in ovarian cancer models also support reciprocal regulation. Oncostatin M receptor signaling increases STAT3 occupancy at the promoters of ITGAV, ITGB3 and ITGB8, whereas disruption of RGD-dependent integrin interactions in cells cultured on fibronectin reduces STAT3 Tyr705 phosphorylation [48]. Combined interference with the OSMR and integrin pathways also increases cisplatin sensitivity. These findings indicate that cytokine-induced integrin expression and ECM-dependent signaling can cooperate to maintain STAT3 activity, linking inflammatory adaptation to adhesion-dependent drug resistance.

NF-κB family members link transcriptional adaptation to the production and sensing of extracellular niches. In PDAC, c-Rel directly regulates FN1 and ITGB3 transcription, supporting fibronectin-rich niches, resistance to isolation stress and metastatic competence [11]. A mechanism linking ECM signals to NF-κB activation has been described in ovarian cancer, where periostin activates ERK/NF-κB signaling through β3-and β5-containing integrins. This pathway promotes cytokine and chemokine production associated with monocyte recruitment and M2-like macrophage polarization, extending the effects of matrix signaling beyond tumor cells [49]. Across these models, NF-κB family signaling operates on both sides of ECM–integrin regulation: c-Rel drives ECM and integrin expression in PDAC, whereas periostin–integrin signaling activates NF-κB in ovarian cancer.

➢ Integrated transcriptional network and ECM–integrin signaling

The above findings suggest a model in which ECM–integrin signaling helps coordinate three adaptive requirements: nutrient and redox homeostasis, access to supportive extracellular niches, and survival when a dominant pathway is constrained. Direct links between transcriptional hubs provide one mechanism of convergence. During inhibition of glutaminolysis, YAP induces ATF4 and Sestrin2, coupling nutrient stress to mTORC1 restraint and cell survival [50]. In melanoma cells, ATF4 increases NRF2 transcription and induces the glutathione-degrading enzyme CHAC1, thereby sustaining NRF2 activation [51]. These connections support cooperation between nutrient sensing and antioxidant defense, although a continuous integrin–YAP–ATF4–NRF2 cascade has not been established in a single model. Parallel circuits converge on extracellular support: YAP-dependent CTGF secretion and fibroblast ATF4 promote matrix production, STAT3 and c-Rel increase selected integrin or ECM transcripts, and integrin β1–Piezo1 signaling stabilizes HIF-1α to support angiogenic outputs [44], [46]. Their common feature is the capacity to modify the conditions that permit continued adaptation, rather than the obligatory activation of every transcriptional hub.

The relative contributions of these modules depend on matrix mechanics, cell identity and the initiating stress. For example, soft matrix conditions can favor NRF2-dependent protection in disseminated breast cancer cells, demonstrating that adaptive survival need not require a uniformly high-tension state [30]. Compensation becomes particularly evident after therapeutic perturbation: CAF-derived phosphatidylcholines activate tumor-cell JAK2–STAT3 signaling during FAK inhibition in esophageal squamous cell carcinoma, whereas TEAD–YAP blockade can engage a VGLL3–SOX4–PI3K–AKT survival response in cancer models [52]. These findings describe experimentally supported bypass mechanisms during treatment but do not establish that all six transcriptional programs replace one another during nutrient deprivation. Together, these findings suggest a context-dependent network in which partially overlapping survival outputs and reciprocal extracellular remodeling provide multiple routes to persistence. Testing this model will require time-resolved perturbation of individual and combined nodes, with tumor-cell and stromal responses measured separately.

### 3.3 ECM–integrin-dependent transcriptional plasticity drives malignant states

One biological consequence of ECM–integrin transcriptional adaptation is the emergence of flexible tumor cell states [53]. These states are not mutually exclusive but represent different manifestations of stress tolerance, including stemness, dormancy, immune evasion, metastasis and drug tolerance [54], [55], [56] (Fig. 3).

**Fig. 3:**
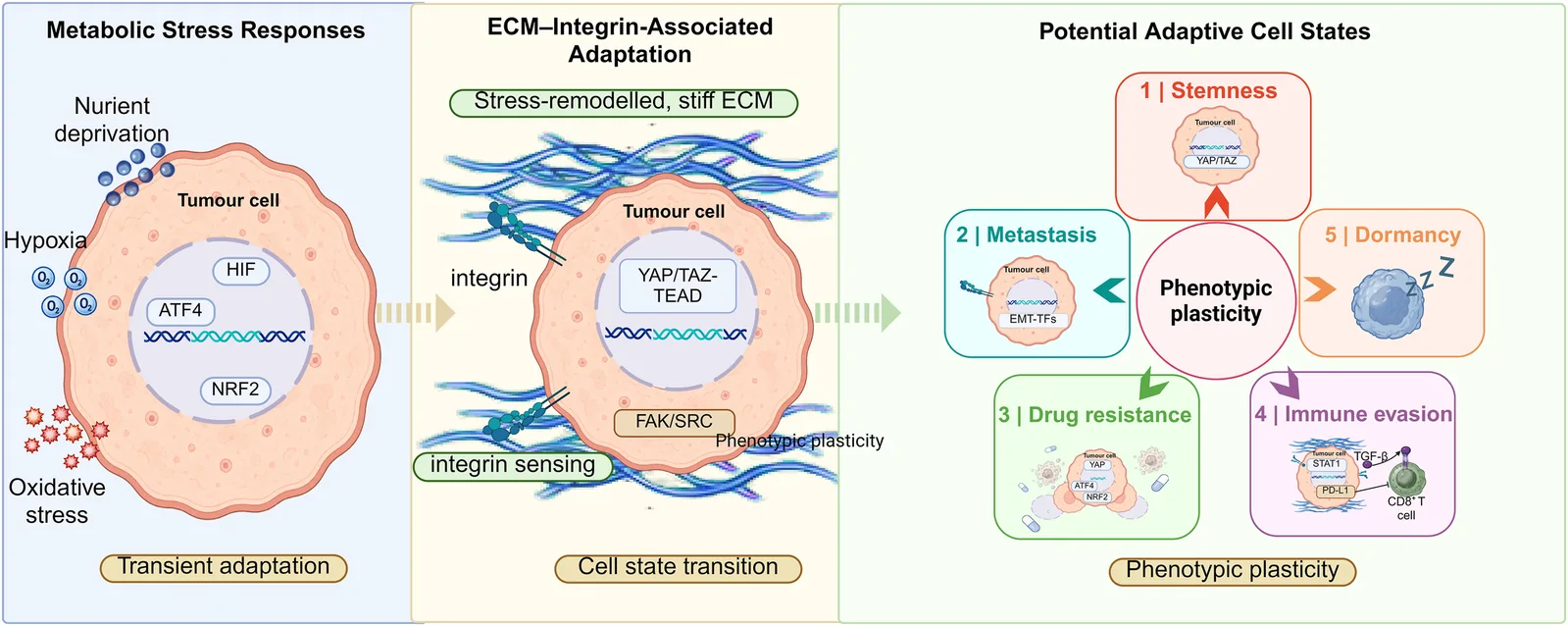
**Context-dependent contributions of ECM–integrin signaling to transcriptional plasticity and tumor evolution.** The schematic illustrates three interconnected processes rather than a fixed sequential pathway. Microenvironmental stresses, including hypoxia, nutrient deprivation and oxidative stress, can activate HIF-, ATF4- and NRF2-associated transcriptional programs that support tumor-cell adaptation. In parallel or under evolving microenvironmental conditions, changes in ECM composition and mechanical properties can modulate integrin-associated signaling, with YAP/TAZ-mediated transcriptional regulation illustrated as one potential mechanism contributing to cell-state plasticity. Depending on the tumor and microenvironmental context, these stress-responsive and integrin-associated programs may independently or cooperatively contribute to phenotypic changes associated with stemness, metastatic competence, therapy resistance, and immune evasion. These processes represent potentially interconnected and context-dependent mechanisms rather than an obligatory sequence of events during tumor evolution.

Integrin-dependent adaptation can preserve tumor-initiating competence under nutrient deprivation or isolation stress. In lung cancer cells, αvβ3 supports AMPK–PGC-1α-dependent metabolic plasticity, whereas PDAC cells respond to isolation stress through LPAR4–AKT–CREB-dependent formation of fibronectin-rich niches [9], [10]. Transcriptional regulation provides additional routes to this phenotype. In PDAC, hypoxia and inflammatory cytokines induce STAT3-dependent ITGB3 expression, increasing stress tolerance and tumor initiation [19]. In high-grade serous ovarian cancer, RelB-dependent programs sustain an αvβ3-positive, stress-tolerant cancer stem-like population associated with relapse; combining RelB depletion with αvβ3 inhibition reduces tumor burden and prolongs survival in the reported model [57]. Together, these examples show how metabolic adaptation, extracellular niche construction and integrin expression can support tumor initiation through distinct, context-specific mechanisms.

ECM-dependent survival also supports dormant or drug-tolerant populations, although these states should not be treated as equivalent. Fibronectin assembly involving α5β1 and αvβ3 supports the maintenance of dormant breast cancer cells during nutrient limitation [58]. In disseminated breast cancer models, a soft lung matrix sustains DRP1–NRF2-dependent protection against cisplatin, linking niche mechanics to the survival of cells that can subsequently resume growth [30]. By contrast, following EGFR inhibition in a lung adenocarcinoma model, ILK–SRC family kinase–YAP signaling supports drug-tolerant persistence [59]. In ovarian cancer, OSMR–STAT3-dependent integrin expression and fibronectin-associated signaling contribute to cisplatin resistance [48]. These findings indicate that both reduced-tension niches and active adhesion-associated signaling can sustain treatment-tolerant cells, depending on the cell state and therapeutic pressure.

Integrin-associated programs can facilitate immune escape through both tumor-cell signaling and the regulation of extracellular cytokine activity. Integrin αvβ3 promotes IFN-STAT1-dependent PD-L1 expression in several epithelial cancer cell lines, while αvβ6-mediated TGF-β activation supports an immune-evasive program in triple-negative breast cancer [60], [61]. In a colorectal cancer model, inhibition of αvβ6 reduces tumor-promoting TGF-β activity, increases cytotoxic T-cell responses and improves the efficacy of PD-1 blockade in ITGB6-expressing tumors [62]. These observations extend the role of integrins beyond adhesion to the regulation of immune-cell access and function; the effects depend on the receptor, tumor context and responding cell compartment.

Metastatic competence similarly requires coordination of matrix interactions with stress tolerance. In PDAC, c-Rel-dependent FN1 and ITGB3 expression couples extracellular niche formation to isolation stress resistance and epithelial–mesenchymal plasticity [11]. In radioresistant triple-negative breast cancer, the NRF2–HNRNPL–circular RNA program increases ITGB3 transcript stability and integrin β3 expression, promoting migration and invasion [47]. These mechanisms connect stress-adaptive transcription or RNA regulation to the adhesive machinery required for dissemination. They also distinguish matrix-assisted survival and invasion from a generalized claim that every stress response drives a complete epithelial-to-mesenchymal transition (EMT).

Across tumor types, integrin-dependent sensing of adhesive and mechanical ECM cues may represent a broadly shared organizational principle. In contrast, the dominant ECM ligands, integrin heterodimers, upstream stress signals and downstream transcriptional programs vary according to tumor type and microenvironmental context. For example, LPAR4-driven fibronectin niche formation has been demonstrated in PDAC, αvβ3-dependent metabolic adaptation in lung cancer, and α6β4-associated ferroptosis resistance in breast cancer models. Together, these findings illustrate a shared ECM–integrin adaptive framework with tumor-specific molecular implementations.

Collectively, ECM–integrin signaling can sustain stress-adaptive transcription and tumor-cell plasticity. Persistent adaptive states may reflect either continued dependence on ECM cues or cell-intrinsic epigenetic changes that maintain these phenotypes after those cues are removed.

## 4 Ecosystem-wide adaptation to metabolic stress: remodeling the tumor microenvironment

Tumor adaptation is not sustained solely by cancer-cell-intrinsic mechanisms [1]. Instead, metabolic stress reshapes multiple cellular compartments within the tumor microenvironment (TME), generating a coordinated adaptive ecosystem [63]. Hypoxia, nutrient deprivation, lactate accumulation, and redox imbalance influence tumor cells, cancer-associated fibroblasts (CAFs) and immune cells simultaneously, altering their metabolic states, transcriptional programs, and interactions [64]. These compartment-specific adaptations are interconnected through ECM remodeling, metabolic exchange and immune regulation, ultimately reinforcing tumor-cell plasticity and therapeutic resistance.

### 4.1 Metabolic stress drives CAF state plasticity and ECM remodeling

CAFs represent a central adaptive hub linking metabolic stress, ECM organization and tumor cell behavior [65]. CAFs respond to environmental stress by altering matrix composition, mechanics and signaling output [63], [66]. Under hypoxia, nutrient limitation and redox stress, CAFs reprogram metabolic pathways, stress-responsive transcriptional programs and contractile functions to sustain ECM synthesis and remodeling [63], [67](Fig. 4A).

**Fig. 4:**
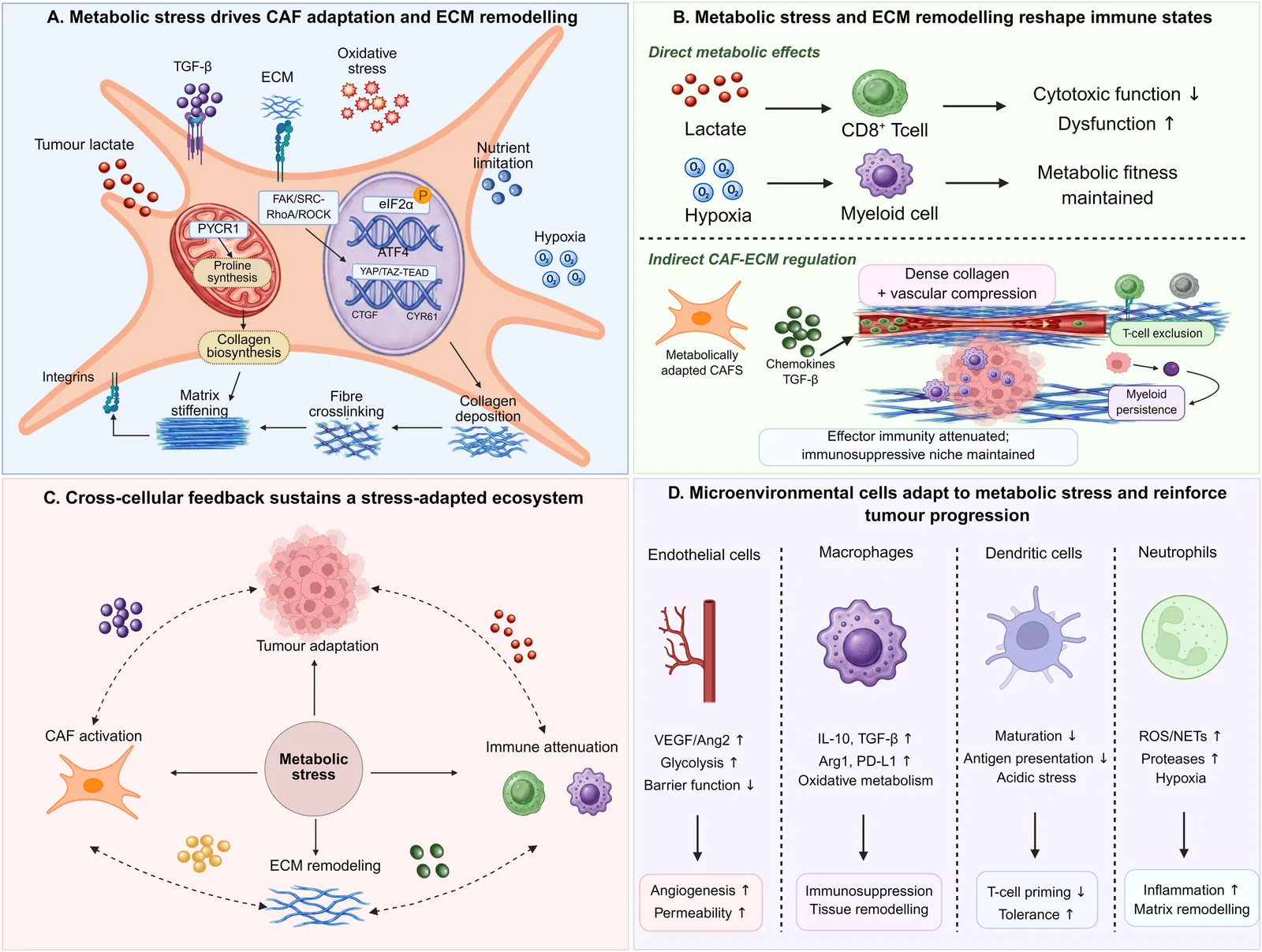
**Context-dependent remodeling of stromal and immune compartments in response to metabolic stress within the tumor microenvironment. A.** Hypoxia, nutrient limitation, oxidative stress and tumor-derived lactate can influence CAF metabolism and mechanotransduction through distinct, potentially interconnected mechanisms. PYCR1-dependent proline synthesis, TGF-β signaling and integrin-associated pathways involving FAK/SRC, RhoA/ROCK, ATF4 and YAP/TAZ may contribute to CAF activation, collagen deposition and matrix remodeling in a context-dependent manner. Macropinocytosis may additionally support the maintenance of myCAF identity under nutrient-limited conditions in certain experimental models. **B.** Metabolic stress can compromise CD8^+^ T-cell cytotoxicity while supporting the metabolic adaptation of certain myeloid cell populations. CAF-derived chemokines, TGF-β, remodeled collagen and matrix-associated vascular compression may further influence T-cell infiltration and myeloid cell accumulation through distinct, context-dependent mechanisms. **C.** Tumor-cell adaptation, CAF activation, ECM remodeling and altered immune responses may interact through reciprocal feedback mechanisms that reinforce stress tolerance in certain tumor contexts. Such interactions could also contribute to adaptive compensation following single-target interventions, although their relative contributions remain context-dependent. **D.** In response to metabolic stress, endothelial cells, macrophages, dendritic cells and neutrophils may undergo distinct metabolic and functional adaptations, including pro-angiogenic, immunosuppressive, tolerogenic or inflammatory programs. These heterogeneous responses can contribute to the establishment or maintenance of tumor-supportive microenvironments in specific contexts. Together, these processes illustrate parallel and potentially interconnected mechanisms of stromal and immune adaptation rather than a single, universally established signaling sequence.

The integrated stress response transcription factor ATF4 provides one mechanism linking nutrient stress to stromal adaptation [44]. In fibroblasts, eIF2α–ATF4 signaling promotes amino acid metabolism, collagen synthesis and pro-angiogenic programs under nutrient limitation [44]. CAFs can also maintain ECM production through metabolic flexibility, including the utilization of tumor-derived lactate and PYCR1-dependent proline synthesis to support collagen biosynthesis [44]. Thus, metabolic adaptation enables CAFs to preserve matrix production even within resource-limited tumor niches.

Beyond metabolic support, stress-induced CAF activation reshapes ECM mechanics [40], [68]. Hypoxia and oxidative stress can enhance TGF-β signaling, actomyosin contraction, and collagen crosslinking [5], [63], promoting focal adhesion maturation and integrin–FAK/SRC–Rho signaling [40], [68]. Activated YAP/TAZ further reinforce this process by inducing ECM-associated factors, including CTGF and CYR61, establishing a positive feedback loop in which metabolic stress promotes matrix stiffening, and stiff ECM maintains mechanotranscriptional activation [38], [40].

Importantly, CAF adaptation is highly context dependent. Nutrient stress does not impose a universal CAF phenotype but can redistribute CAF states according to nutrient availability and signaling context [66]. For example, in PDAC, macropinocytosis helps maintain the myofibroblastic CAF (myCAF) phenotype under glutamine limitation. Blocking this nutrient acquisition pathway under the same conditions promotes MEK/ERK-dependent inflammatory CAF (iCAF) reprogramming. In spontaneous mouse PDAC model, macropinocytosis inhibition reduces collagen deposition and increases immune-cell infiltration [69]. Thus, nutrient availability and nutrient acquisition jointly shape CAF state plasticity.

Through these processes, CAFs link metabolic adaptation to changes in ECM composition and mechanics. Fibronectin deposition, collagen crosslinking and matrix stiffening subsequently define the adhesive and mechanical signals sensed by tumor cells through integrins, thereby linking stromal adaptation with tumor-cell transcriptional plasticity.

### 4.2 Metabolic stress and ECM remodeling reshape immune cell states

Immune cells are also directly influenced by the metabolically constrained TME [64]. Effector T cells require substantial metabolic activity to maintain proliferation, migration and cytotoxic function; however, glucose depletion, amino acid competition, hypoxia and lactate-associated acidosis can impair these processes and promote dysfunctional states [25], [64]. In contrast, immunosuppressive populations, including regulatory T cells and certain myeloid subsets, can maintain fitness through alternative metabolic programs, creating selective pressure that favors immune escape [64]. However, sodium lactate under non-acidifying conditions can support CD8^+^ T-cell stemness and antitumor activity in preclinical models, indicating that lactate exposure and acidosis should be distinguished [70] (Fig. 4B).

ECM remodeling further amplifies these effects by controlling immune-cell access and function [71]. Collagen accumulation, matrix contraction and vascular compression restrict immune-cell trafficking while reducing oxygen and nutrient availability [72]. Thus, ECM remodeling can influence immunity not only by creating physical constraints but also by altering local perfusion, oxygenation and nutrient availability, thereby shaping immune-cell localization and functional capacity.

CAF-derived signals contribute substantially to this immune adaptation. Glycolytic CAF populations can retain CD8^+^T cells at tumor margins through chemokine-dependent mechanisms in soft-tissue sarcomas, whereas lactate-driven fibroblast programs promote inflammatory and immunosuppressive niches in oral squamous cell carcinoma [73], [74]. In addition, ECM-associated receptors directly regulate immune responses, as integrin-dependent activation of latent TGF-β and collagen receptor signaling can suppress cytotoxic T-cell activity and contribute to resistance to immune checkpoint blockade [75], [76].

Nevertheless, the immune consequences of ECM remodeling are context-dependent. Specific CAF states may restrict immunity in some tumors while supporting immune organization in others [77], [78]. Therefore, therapeutic targeting of stromal adaptation requires a deeper understanding of CAF identity, spatial organization and stress context rather than broadly eliminating fibroblast populations.

### 4.3 Cross-cellular feedback sustains a stress-adapted tumor ecosystem

Adaptation within tumor cells, CAFs and immune cells does not occur independently but forms a network of reciprocal interactions at the tissue level [63]. Tumor-cell metabolism generates environmental pressures, including oxygen depletion, nutrient competition and lactate accumulation, which reshape CAF and immune-cell states [74]. CAFs subsequently remodel ECM architecture, provide metabolic support and generate integrin-active niches that reinforce tumor-cell survival and transcriptional plasticity [79]. Meanwhile, immune dysfunction reduces immune-mediated selective pressure on tumor cells, allowing stress-adapted populations to persist [64] (Fig. 4C).

Beyond CAFs and lymphocytes, metabolic stress also reprograms endothelial and innate immune compartments. Enhanced endothelial glycolysis supports abnormal tumor vessel growth. In a glioblastoma model, ATF4-induced phosphoglycerate dehydrogenase (PHGDH) expression supports glutathione synthesis and redox balance, sustaining endothelial glycolysis and excessive sprouting [80], [81]. Separately, endothelial ANGPTL4 deletion reprograms lipid–glucose metabolism, stabilizes vascular structures and reduces permeability [82]. In glioblastoma-associated monocyte-derived macrophages, PERK–ATF4 signaling promotes glycolysis and histone lactylation, increasing IL-10 expression [83], while PHGDH-dependent serine synthesis sustains mitochondrial respiration, α-ketoglutarate–mTORC1 signaling and the expression of ARG1, TGF-β, and PD-L1 [84]. Tumor-derived lactate similarly impairs dendritic-cell cross-presentation and T-cell priming [85] and, through SREBP2, drives CD63-positive regulatory dendritic-cell maturation and immune tolerance [86]. In a brain tumor model, hypoxia-driven glycolysis and histone lactylation promote the immunosuppressive activity of CD71-positive neutrophils [87]. In a separate inflammatory setting, neutrophil extracellular trap (NET)-associated elastase and MMP9 cleave laminin, promoting the reactivation of dormant cancer cells in mice [88]. Together, these compartment-specific adaptations can contribute to coordinated angiogenic, immunosuppressive and matrix-remodeling programs that reinforce a stress-adapted tumor ecosystem (Fig. 4D).

The relatively slow turnover of some ECM components provides a potential basis for prolonged extracellular signaling [13]. Changes in collagen deposition, fiber alignment and crosslinking may help sustain integrin–FAK/SRC–cytoskeletal activity, linking ECM remodeling to the maintenance of adaptive responses [72].

Collectively, metabolic stress can reshape programs in tumor cell, CAFs, and immune cells, alter ECM composition and mechanics, and influence integrin-associated signaling and transcriptional plasticity. Rather than following a fixed sequence, these processes may operate in parallel and interact through context-dependent feedback among tumor cells, CAFs, the ECM and immune cells. Although several of these interactions have been demonstrated in specific experimental models, their integration into a self-reinforcing adaptive network remains to be fully established. Such potential feedback and compensatory mechanisms may partly explain the limited durability of therapies targeting individual metabolic or stress-responsive pathways, providing a rationale for exploring therapeutic strategies that simultaneously address metabolic adaptation, stromal remodeling and adaptive signaling programs.

## 5 Therapeutic strategies targeting the metabolic stress–ECM–integrin adaptation network

The metabolic stress–ECM–integrin axis represents a hierarchical adaptive network rather than a single therapeutic target [63]. This network perspective suggests that durable intervention may require coordinated targeting of more than one adaptive node in selected tumor contexts, although the optimal combinations and therapeutic windows remain to be established (Fig. 5). Importantly, therapeutic strategies should be guided by the dominant adaptive dependency within each tumor rather than by the indiscriminate inhibition of stress-responsive pathways [89].

**Fig. 5:**
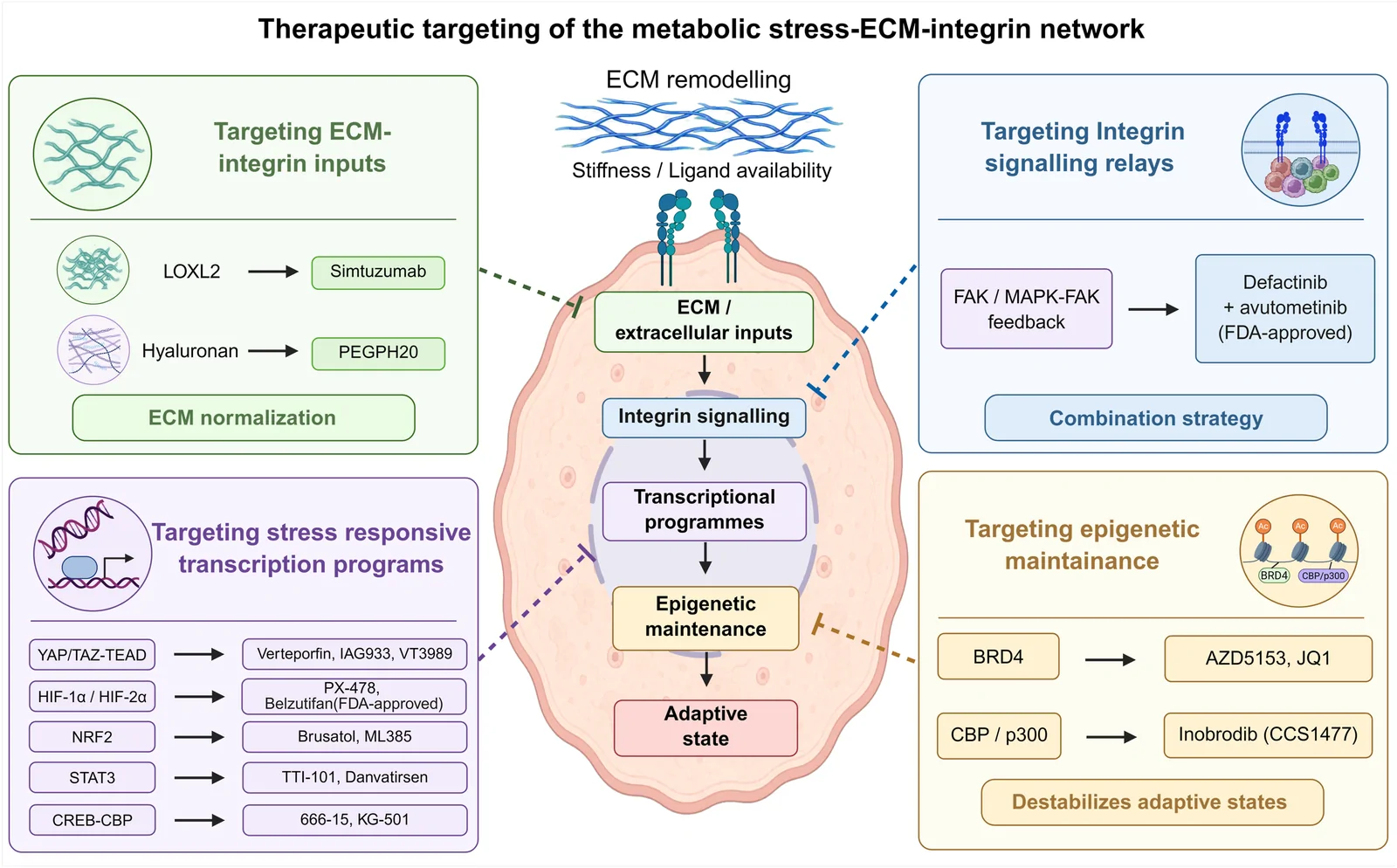
**Therapeutic targeting of the metabolic stress–ECM–integrin adaptation network.** The central schematic illustrates a conceptual framework in which ECM remodeling, integrin-associated signaling, stress-responsive transcriptional programs and chromatin-associated regulatory mechanisms may contribute to tumor adaptation through distinct, potentially interconnected and context-dependent processes rather than a fixed hierarchical signaling cascade. Potential therapeutic strategies target different components of this network, including ECM remodeling, integrin-associated signaling, stress-responsive transcriptional regulation and chromatin-associated mechanisms. ECM-directed approaches include LOXL2 targeting with simtuzumab and hyaluronan degradation with PEGPH20, although these agents have not demonstrated clinical benefit in the settings tested. Integrin-associated and MAPK signaling may be targeted through combined FAK and RAF/MEK inhibition with defactinib plus avutometinib. Transcriptional regulators, including YAP/TAZ–TEAD, HIF, NRF2, STAT3 and CREB–CBP, represent additional potential intervention points, with belzutifan specifically targeting HIF-2α. Chromatin-associated regulation may be targeted through BRD4/BET inhibition with agents such as AZD5153 and the preclinical probe JQ1, or through CBP/p300 bromodomain inhibition with inobrodib. The defactinib-avutometinib combination and belzutifan have each received FDA approval for specific oncology indications, but these approvals do not establish the clinical efficacy of broadly targeting ECM–integrin-dependent stress adaptation. The remaining agents represent investigational, experimental or unsuccessful clinical approaches with varying levels of evidence. Detailed regulatory status, clinical indications, supporting evidence and limitations are summarized in Table 1. The schematic highlights potential intervention points rather than a universally established signaling sequence or validated therapeutic strategy.

### 5.1 Targeting ECM–integrin inputs

The ECM constitutes a major upstream determinant of integrin engagement and signaling [7]. Strategies targeting aberrant matrix remodeling, including those aimed at inhibiting collagen crosslinking, reducing hyaluronan accumulation or blocking ligand–integrin interactions, aim to reduce mechanical stress, improve drug delivery and restore immune accessibility [6], [7], [90]. Clinical trials have nevertheless shown limited benefit from these approaches (Table 1). Simtuzumab did not improve progression-free or overall survival when added to gemcitabine in metastatic pancreatic cancer. Similarly, PEGPH20 added to nab-paclitaxel–gemcitabine did not improve either endpoint in hyaluronan-high metastatic pancreatic cancer. The PEGPH20 result illustrates that enrichment for the targeted matrix component does not necessarily predict clinical benefit [91], [92].

These clinical and preclinical observations support a shift in emphasis from indiscriminate stromal depletion toward context-specific functional ECM normalization. In pancreatic cancer models, intact type I collagen restrains tumor growth, whereas proteolytically cleaved collagen promotes it [93]. Identification of dominant ECM ligands, integrin activation states and spatially restricted matrix niches may enable more selective intervention. Short-term matrix modulation combined with chemotherapy, targeted therapy or immunotherapy may provide a more favorable therapeutic window than sustained systemic ECM disruption.

### 5.2 Targeting integrin-associated signaling relays

FAK/SRC signaling provides a direct connection between ECM engagement and intracellular adaptation [94]. Rather than acting solely as a proliferative pathway, FAK functions as an adaptive signaling amplifier that integrates adhesion, mechanical stress and survival signals. Although FAK inhibitors have generally shown limited single-agent activity, defactinib combined with the RAF/MEK inhibitor avutometinib has shown activity in recurrent low-grade serous ovarian cancer. The combination received accelerated FDA approval in 2025 for adults with KRAS-mutated disease after prior systemic therapy (Table 1) [95], [96].

Integrin signaling is highly redundant, allowing alternative adhesion receptors and growth factor pathways to bypass individual nodes [7]. In an esophageal squamous cell carcinoma model, CAF-derived phosphatidylcholines activate tumor-cell JAK2–STAT3 signaling and confer resistance to FAK inhibition [95], [96]. Integrin activation, matrix characteristics and adaptive signaling signatures should therefore be evaluated as candidate stratification markers.

### 5.3 Targeting transcriptional execution of stress adaptation

#### 5.3.1 Stress-responsive transcriptional programs provide additional opportunities for therapeutic intervention

YAP/TAZ–TEAD signaling is a major mechanotranscriptional module linking ECM stiffness to gene expression [40]. In a phase I/II trial, the TEAD inhibitor VT3989 has shown antitumor activity in patients with mesothelioma, supporting the clinical targeting of YAP–TEAD signaling [97]. Therapeutic development must also account for the physiological roles of YAP/TAZ in tissue repair and compensatory signaling, including PI3K–AKT activation observed after TEAD inhibition in preclinical models [97] (Table 1).

Other stress-responsive pathways provide additional opportunities. ATF4 supports adaptation to nutrient limitation and proteotoxic stress, whereas HIF and NRF2 maintain survival under hypoxic and oxidative conditions [39], [98]. However, systemic inhibition of HIF or NRF2 signaling has achieved limited success in cancer therapy (Table 1), largely because these pathways are also essential for maintaining normal tissue homeostasis. These physiological functions restricts the therapeutic window and increases the risk of toxicity [98]. Future approaches will likely require transient, tumor-selective, or microenvironment-specific targeting strategies, combined with treatments that selectively exploit tumor stress dependencies [99].

STAT3 and CREB represent additional stress-adaptive transcriptional regulators that link inflammatory or microenvironmental stress to ECM remodeling and tumor persistence [19], [48]. Although STAT3 inhibitors such as TTI-101 and danvatirsen have entered clinical evaluation, clinical benefits remain context-dependent, highlighting challenges including pathway redundancy, delivery limitations and effects on normal immune functions (Table 1). CREB-targeting inhibitors remain largely at the preclinical stage, with limited clinical translation owing in part to the broad physiological functions of CREB and its interactions with shared transcriptional coactivators (Table 1). Therefore, pharmacodynamic monitoring of phospho-STAT3 and phospho-CREB, alongside ECM-associated outputs, will be essential for defining responsive contexts.

### 5.4 Targeting epigenetic maintenance of adaptive states

Metabolic stress and ECM–integrin signaling can influence chromatin accessibility and enhancer activity, providing a route through which adaptive transcriptional programs may be sustained [8], [100]. Chromatin regulators such as BRD4 and CBP/p300 provide shared platforms that maintain transcriptional programs driven by YAP/TAZ, CREB or other stress-responsive factors [101], [102].

Targeting these epigenetic regulators may therefore weaken the transcriptional programs that support stress adaptation [103]. In a phase I study, AZD5153 showed peripheral pharmacodynamic evidence of target engagement but no objective responses as monotherapy; thrombocytopenia and diarrhea were dose-limiting toxicities. The CBP/p300 bromodomain inhibitor inobrodib is undergoing clinical development primarily in hematological malignancies, including a phase II combination study in relapsed or refractory multiple myeloma (Table 1). However, because these regulators are broadly required for normal transcription, systemic inhibition is associated with toxicity [102], [104]. Future strategies should focus on intermittent dosing, tumor-directed delivery and biomarker-guided patient selection based on stress-associated enhancer activity.

Overall, the therapeutic goal should not be the complete suppression of stress responses, which may be biologically unrealistic, but rather the disruption of the adaptive circuits that allow tumor cells to sense and exploit persistent or recurrent microenvironmental stress. Combining ECM–integrin targeting with metabolic intervention, immunotherapy or epigenetic modulation may provide a rational strategy to overcome tumor plasticity and improve therapeutic durability.

Table 1.

**Table 1:** Representative agents and strategies targeting nodes of the metabolic stress–ECM–integrin adaptation network and their translational status.

Target	Agent or strategy	Network position	Developmental/regulatory status	Mechanistic rationale and potential advantages	Clinical limitations and possible reasons for failure	Reference
LOXL2–collagen crosslinking	Simtuzumab	ECM crosslinking and mechanical input	Phase II completed; evaluated with gemcitabine in untreated metastatic pancreatic adenocarcinoma; no improvement in PFS or OS; did not proceed to phase III	Designed to reduce collagen crosslinking and the stability of the fibrotic matrix	LOXL2 inhibition alone is insufficient to reverse a complex ECM; other LOX-family members and crosslinking pathways may compensate	[91]
Hyaluronan–interstitial pressure	PEGPH20	ECM pressure and perfusion input	Phase III HALO-301 completed (NCT02715804); evaluated in HA-high, untreated metastatic PDAC; gemcitabine–nab-paclitaxel combination did not improve PFS or OS; oncology development discontinued	Reduces hyaluronan burden, interstitial pressure and vascular compression	HA-high stratification did not confer a survival benefit; thromboembolic risk; extensive matrix degradation does not necessarily restore effective delivery	[92]
FAK and MAPK–FAK feedback	Defactinib + avutometinib	Integrin-proximal signal transmission	Received FDA accelerated approval in 2025 for KRAS-mutant recurrent LGSOC after prior systemic therapy; phase III RAMP-301 recruiting (NCT06072781); phase Ib/IIa RAMP-205 in metastatic PDAC ongoing but not recruiting (NCT05669482)	Mechanistically complementary in tumors in which MAPK inhibition induces FAK-dependent feedback	Regulatory approval and established clinical benefit currently apply to KRAS-mutant recurrent LGSOC; activity in other pFAK-high or matrix-stiffened tumors may vary with tumor context, while FAK scaffolding functions and stromal paracrine compensation may persist.	[96]
YAP/TAZ-related phenotypes	Verteporfin	Mechanotranscription proof-of-concept	Approved for ophthalmic photodynamic indications; used primarily for preclinical repurposing and mechanistic validation in oncology; no confirmatory oncology evidence for specific YAP–TEAD inhibition	Supports preliminary assessment of YAP/TAZ dependency	Photodynamic effects, proteostatic disruption and pleiotropic activity complicate attribution of experimental effects specifically to YAP–TEAD inhibition	[38]
YAP/TAZ–TEAD interface	IAG933	Mechanotranscriptional execution	Phase I ongoing but not recruiting (NCT04857372); evaluated in malignant mesothelioma and advanced solid tumors with NF2, LATS1/2 loss or YAP/TAZ fusions; no registered results or mature peer-reviewed clinical efficacy data	Directly disrupts the YAP/TAZ–TEAD protein–protein interaction;	The responsive population, long-term safety and effects on normal-tissue homeostasis remain undefined	[105]; NCT04857372
TEAD palmitoylation	VT3989	Mechanotranscriptional execution	Phase I/II recruiting (NCT04665206); evaluated in mesothelioma, NF2-altered and other refractory solid tumors; expanded to multiple combination cohorts	Provides human proof-of-concept for pharmacological targeting of the Hippo–YAP–TEAD pathway	Proteinuria/albuminuria and peripheral edema; compensatory PI3K–AKT signaling and physiological TEAD functions may constrain the therapeutic window	[97]; NCT04665206
HIF-1α	PX-478	Hypoxia-adaptation buffering	Phase I completed (NCT00522652) in advanced solid tumors and lymphoma; no subsequent confirmatory development	Affects HIF-1α transcription, translation and protein stability	Non-uniform mechanism, limited selectivity, and absence of clear molecular stratification and reliable pharmacodynamic biomarkers	[106]; NCT00522652
HIF-2α	Belzutifan	Genetically defined hypoxia adaptation	Approved for VHL disease-associated RCC, CNS hemangioblastomas and pNETs, and for advanced ccRCC previously treated with PD-1/PD-L1 inhibitors and VEGF–TKIs; indications further expanded in 2025–2026	Clear VHL–HIF-2 genetic dependency and an established basis for patient stratification	Clinical benefit is established in genetically defined VHL–HIF-2-dependent settings; anemia and hypoxia are the principal mechanism-related toxicities.	[107]; NCT03401788; NCT04195750
NRF2	Brusatol; ML385	Redox buffering	Preclinical; no oncology clinical trials for either agent as of the search date	May expose the redox dependency of tumors with KEAP1 inactivation or sustained NRF2 activation	Brusatol suppresses global protein synthesis; the selectivity, pharmacokinetics and normal-tissue toxicity of ML385 remain unresolved; prolonged inhibition may impair normal tissues and immune cells	[39], [108], [109]
STAT3	TTI-101	Stromal and therapy-induced compensatory feedback	Phase I completed (NCT03195699) in multiple refractory solid tumors; subsequent phase Ib/II NCT05440708 in HCC recruiting	Directly suppresses STAT3 activity; potentially suited to tumors with active IL-6/JAK signaling or STAT3 compensation after FAK inhibition	Early single-arm signals require validation in randomized studies; STAT-family redundancy and effects on normal immune function constrain development	[110]; NCT05440708
STAT3 mRNA	Danvatirsen (AZD9150)	Stromal inflammation and immune compensation	Phase Ib/II SCORES completed (NCT02499328); randomized phase II PEMDA-HN did not improve ORR (NCT05814666); phase I study in relapsed/refractory AML or MDS recruiting (NCT05986240)	Antisense oligonucleotide reduces expression of STAT3 that is conventionally difficult to drug and can be combined with immunotherapy	Tumor-tissue delivery, pharmacokinetics and tumor-type dependence are substantial; clinical benefit in solid tumors has been inconsistent	[111], [112]
CREB–CBP	666-15; KG-501	Transcriptional execution of stress-to-ECM output	Preclinical; no oncology clinical trials for either agent as of the search date	Used to interrogate stress-induced ECM-production programs	CREB contributes to neural, metabolic and immune homeostasis; selectivity and in vivo pharmacology remain insufficient for direct therapeutic translation	[10], [101], [113]
BRD4	AZD5153; JQ1	Enhancer-state recognition and maintenance	JQ1 is a preclinical tool; AZD5153 phase I completed (NCT03205176) in relapsed/refractory solid tumors and lymphoma and evaluated with olaparib; limited antitumor activity and no confirmatory late-stage development	Attenuates dependence on acetylated chromatin shared by YAP/TAZ and other stress-responsive transcription factors	Thrombocytopenia, diarrhea and global transcriptional toxicity limit dosing; stromal cytokines may activate bypass signaling	[103], [104]
CBP/p300 co-activation layer	Inobrodib (CCS1477)	Shared transcriptional co-activation and chromatin maintenance	Solid-tumor phase I/IIa NCT03568656 completed without registered results; hematological-malignancy phase I/IIa NCT04068597 recruiting; phase II DoMMino-1 in relapsed/refractory MM recruiting (NCT07096778)	Targets co-activator dependency shared by CREB, AR, MYC and other transcription factors	Limited evidence in solid tumors; hematological toxicity may constrain combination dosing; current clinical evidence is concentrated in hematological malignancies, with limited data in ECM-rich solid tumors.	NCT03568656; NCT04068597; NCT07096778

## 6 Conclusions and future perspectives

Although emerging evidence suggests that metabolic stress and ECM–integrin remodeling are functionally interconnected in cancer, the underlying molecular mechanisms and their contributions to tumor adaptation remain incompletely understood. Key unresolved questions include how distinct metabolic stresses reshape ECM composition and mechanical properties, how these changes are sensed and transmitted through specific integrins, and to what extent the resulting transcriptional adaptations are reversible or sustained. Moreover, the causal relationships linking metabolic stress, ECM–integrin signaling and transcriptional plasticity remain insufficiently established, particularly in physiologically relevant tumor contexts.

These unresolved questions extend beyond tumor-cell-intrinsic mechanisms to encompass reciprocal interactions among tumor cells, CAFs, immune cells and the ECM. Tumor-derived metabolites, cytokines, extracellular vesicles and matrix-remodeling factors can influence stromal and immune-cell states, while CAFs and immune cells, in turn, modulate nutrient availability, ECM organization and inflammatory signaling. These reciprocal interactions contribute to spatially heterogeneous tumor niches that may influence tumor persistence, metastasis and therapeutic resistance. However, their relative contributions and underlying mechanisms are likely to vary with stromal composition, metabolic conditions and tumor context.

Future studies should define how YAP/TAZ, ATF4, HIF, NRF2 and related regulators cooperate or compensate for one another across tumor contexts. Their activity should be examined in relation to ECM composition, integrin activation, redox state, chromatin regulation and treatment history. Temporal studies should distinguish continued ECM-dependent signaling from cell-intrinsic epigenetic stabilization. Combining longitudinal single-cell and spatial profiling with stress withdrawal and functional perturbation would help determine which adaptive states are reversible and which dependencies sustain persistent responses.

An important limitation of the current evidence is that many proposed links among metabolic stress, ECM remodeling, integrin signaling and transcriptional plasticity have been established in cell-culture systems or selected mouse models. Human tumors additionally contain complex spatial metabolic gradients, stromal heterogeneity and treatment histories that are difficult to reproduce experimentally. Future validation will therefore benefit from patient-derived models that preserve multicellular interactions and ECM architecture, together with longitudinal human specimens and spatial multi-omic analyses.

Therapeutically, these concepts suggest that targeting individual metabolic enzymes or stress-responsive transcription factors alone may be insufficient because tumors can exploit pathway redundancy, spatial heterogeneity and compensatory adaptation. Combinations of extracellular, integrin-associated and transcriptional interventions should be guided by tumor-specific dependencies and treatment tolerability. Matrix-directed approaches should prioritize functional ECM normalization while preserving protective stromal functions. ECM architecture, integrin activation, transcriptional states and spatial metabolic features are candidate measures for prospective patient-stratification studies.

Single-cell multi-omics, spatial profiling, patient-derived organoid or explant models and longitudinal clinical sampling provide complementary approaches for defining stress-responsive ECM niches and their temporal evolution in human tumors. Ultimately, the goal is to identify the dominant metabolic stress–ECM–integrin dependencies within individual tumors and use them to guide selective therapeutic intervention.

## CRediT authorship contribution statement

**Zixing Meng:** Writing – review & editing, Writing – original draft, Conceptualization. **Xin He:** Writing – review & editing, Writing – original draft, Conceptualization. **Mengyang Li:** Writing – review & editing, Writing – original draft. **Chengsheng Wu:** Writing – review & editing, Writing – original draft, Supervision, Project administration, Funding acquisition, Conceptualization.

## Funding

This work was supported by the 10.13039/501100001809National Natural Science Foundation of China (82573393, C.W.), Beijing Natural Science Foundation (L2611018, C.W.), the Research Foundation of Chinese Academy of Sciences (C.W.), Tanji Young Talents Grant (C.W.) and the Start-up Fund from the University of Chinese Academy of Sciences (C.W.).

## Declaration of competing interest

The authors declare that they have no known competing financial interests or personal relationships that could have appeared to influence the work reported in this paper.

## Data Availability

No data was used for the research described in the article.
